# Deep Reinforcement Learning for Articulatory Synthesis in a Vowel-to-Vowel Imitation Task

**DOI:** 10.3390/s23073437

**Published:** 2023-03-24

**Authors:** Denis Shitov, Elena Pirogova, Tadeusz A. Wysocki, Margaret Lech

**Affiliations:** 1 School of Engineering, RMIT University, Melbourne 3000, Australia; 2 Department of Electrical and Computer Engineering, University of Nebraska-Lincoln, Lincoln, NE 68588, USA; 3 Faculty of Telecommunications, Computer Science and Electrical Engineering, Bydgoszcz University of Science and Technology, 85-796 Bydgoszcz, Poland

**Keywords:** speech modeling, reinforcement learning, speech synthesis

## Abstract

Articulatory synthesis is one of the approaches used for modeling human speech production. In this study, we propose a model-based algorithm for learning the policy to control the vocal tract of the articulatory synthesizer in a vowel-to-vowel imitation task. Our method does not require external training data, since the policy is learned through interactions with the vocal tract model. To improve the sample efficiency of the learning, we trained the model of speech production dynamics simultaneously with the policy. The policy was trained in a supervised way using predictions of the model of speech production dynamics. To stabilize the training, early stopping was incorporated into the algorithm. Additionally, we extracted acoustic features using an acoustic word embedding (AWE) model. This model was trained to discriminate between different words and to enable compact encoding of acoustics while preserving contextual information of the input. Our preliminary experiments showed that introducing this AWE model was crucial to guide the policy toward a near-optimal solution. The acoustic embeddings, obtained using the proposed approach, were revealed to be useful when applied as inputs to the policy and the model of speech production dynamics.

## 1. Introduction

Starting from early studies of simulating the human vocal tract [1,2,3], the research aimed to develop a computational model of the vocal tract that could synthesize a natural human-like sound. Over the last decades, there has been significant progress in designing fine-detailed complex models [4,5,6,7]. Recently published studies [7] achieved a high quality of naturalness of the synthesized speech. However, this improvement was accompanied by an increasing number of parameters that needed to be carefully controlled during the synthesis process. To address this issue, a number of different approaches for controlling a vocal tract have been reported in [8,9,10]. Most of them apply a rule-based algorithm where gestures and transitions between the different vowels and consonant shapes follow the user-defined rules.

However, according to to [8], phonemes in natural speech are not static and affect the articulation of its neighboring sounds within or even across word boundaries, with articulatory movements constantly overlapping each other. Authors of [11] proposed a dynamic description of speech gesture and a task-dynamic model to coordinate between different articulators and the coarticulation between subsequent gestures based on those predefined goals. Even though the concept of gestures helps to reduce the problem of articulatory control, utterance generation still requires thorough manual work, which includes defining unknown parameters of gestures and their timings.

One approach to avoiding hand-crafting the rules and gesture timing for vocal tract control is to apply supervised learning to a problem [12,13]. In [14], authors trained a long short-term memory (LSTM) regression model to perform an inverse mapping from an acoustic to an articulatory space. This approach, however, still relies on obtaining training data using the rule-based method. Alternatively, the acoustic-to-articulatory mapping can be obtained using reinforcement learning through episodic interaction with the environment, i.e., the vocal tract. Recent studies in reinforcement learning have shown a remarkable advancement in various planning and control tasks [15,16,17,18]. Especially promising is the progress made in the field of model-based reinforcement learning. In [19], authors train a generative recurrent neural network to model several environments and then use this model for learning the policy. The most valuable advantage of the model-based methods is that the learning process is usually more sample-efficient compared to its model-free counterpart as shown in [18,20]. Given that current articulatory models for speech synthesis are computationally intensive, sample efficiency becomes one of the most important factors to consider when developing a learning algorithm. However, according to [18], model-based approaches impose other difficulties such as model inaccuracies, accumulating errors of multi-step predictions, and overconfident predictions outside of the training distribution.

This study aims to evaluate the applicability of model-based reinforcement learning in the domain of articulatory speech synthesis. It proposes a new learning algorithm for articulatory imitation synthesis. A vowel-to-vowel imitation task has been chosen as the first approximation of general speech imitation. However, the proposed learning algorithm is not constrained to the vowel-to-vowel scenario and its applicability to general speech imitation will be a subject of our future research.

The following Section 2 provides a definition of the vowel-to-vowel imitation task along with the description of the articulatory synthesizer which is considered to be a part of the learning problem to be solved.

### Contributions

As a major contribution, this study proposes an algorithm for learning the policy via utilizing a neural network approximating the vocal tract model. This neural network and the policy are trained in the same loop with their own objectives to predict the vocal tract. The acoustic state transitions are provided as the action input. The algorithm does not require external training data since both the policy and the model approximation are learned through interactions with the actual vocal tract model. Another advantage of the algorithm is its sample efficiency, which significantly reduces the number of these computationally costly interactions. To stabilize the training, early stopping [21] was incorporated into the algorithm.

Additionally, the study proposes to extract acoustic features using the acoustic word embedding (AWE) model [22]. This model was trained to discriminate between different words and allows for compact encoding of acoustics while preserving contextual information of the input. Our preliminary experiments showed that the AWE model was crucial to guide the policy towards a near-optimal solution, where the optimal solution would be the policy that can produce the exact copy of the reference sound.

## 2. Vowel-to-Vowel Imitation Task

The goal of the speech imitation task is to produce a speech sound that is perceptually identical to the reference speech. In the context of articulatory synthesis, this goal can be reformulated to find the policy that controls the vocal tract in a way so that the sound produced by the synthesizer can match the reference sound. Following the task formulation, the reference sound is included as the input to the policy. In addition, information about the synthesizer’s vocal tract shape and the synthesized speech is also fed as policy inputs. The output of the policy is an action that changes the vocal tract shape and is then used by the synthesizer to produce the sound. The following Section 2.1 and Section 2.2 discuss the synthesizer employed in this study and describe the corresponding vocal tract state space along with the action space.

### 2.1. Articulatory Speech Synthesis Model

The speech production model for this study was chosen from a variety of articulatory synthesizers such as VocalTractLab (VTL) [7], ArtiSynth [23], Maeda’s articulatory model [2], and CASY [24]. The VTL was selected due to the high naturalness of the synthesized speech. However, it should be noted that the proposed algorithm for learning the policy is not bound to the VTL and, in principle, can be combined with any synthesizer.

**Figure 1:**
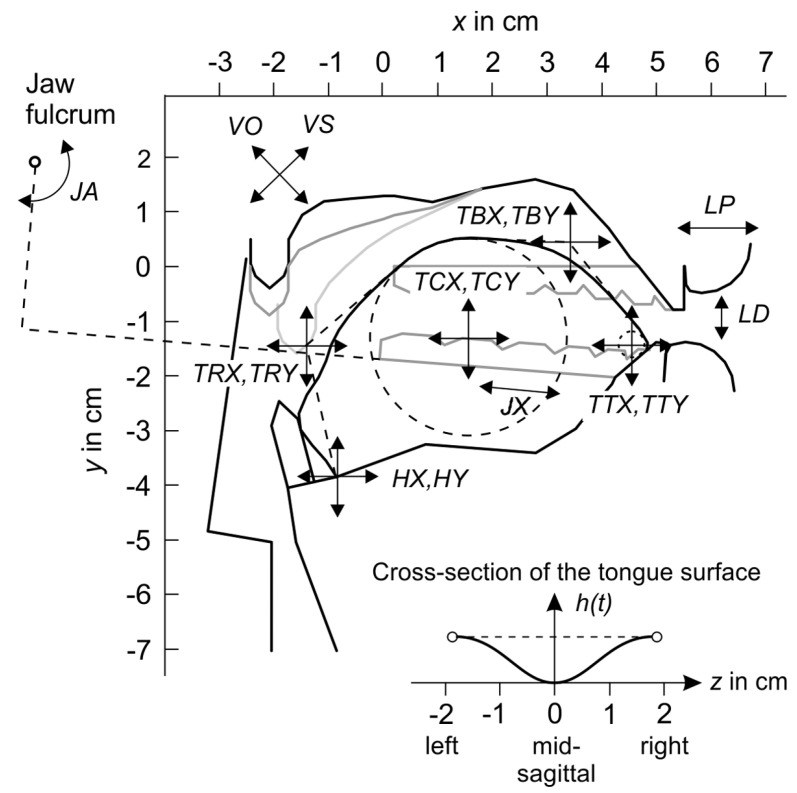
Areas of influence of the vocal tract parameters (adapted from [7]).

One of the VTL key components is the 3D vocal tract model, which represents the time-varying shape of the supra-glottal airways. This 3D shape is the basis for the accurate calculation of values for the area functions of the vocal tract model used to simulate speech acoustics. Generally, the vocal tract model is defined by the geometry of articulators’ surfaces and vocal tract walls. Their shapes and positions in the 3D space are specified by a set of control parameters, each corresponding to one degree of freedom (DOF), as depicted in Figure 1.

For each of the vocal tract model parameters presented in Table 1, the minimum and maximum values were derived from the MRI images of the real male speaker to prohibit anatomically improbable shapes while maintaining the flexibility needed to produce a relatively large set of speech sounds. It was achieved by taking into account anatomical geometrical constraints which prevent inter-penetrations of the articulators. Having control over the movement of articulators it was possible to make a quantitative specification of the time-varying shapes of the vocal tract. The change in time sequences of the vocal tract shapes was used to calculate values of the articulatory areas that were required to perform acoustic simulations and generate speech waveforms.

### 2.2. Vocal Tract State and Action Spaces

A set of available vocal tract states $S_{vt}$ is given as a continuum of all possible shapes and positions in the 3D space of all articulators within the VTL model. It is important to note that the 23 control parameters listed in Table 1 are continuous. Altogether, these parameters define the 23-dimensional bounded continuous-state space $S_{vt}\to R^{23}$.

To control the vocal tract shape, an action *a* is defined as a vector of transitions of all articulators presented in the VTL model and listed in Table 1. Thus, all possible actions form the 23-dimensional continuous action space $A\to R^{23}$.

## 3. Method

### 3.1. Acoustic Representation

One of the major contributions of this study is the employment of the embedding model for extracting acoustic speech features in the context of articulatory speech synthesis. To our knowledge, there were no attempts to use acoustic embedding models in related works. Most of the current methods suggest conventional acoustic speech features. Authors in [25] encoded auditory targets for their model of speech production using three pairs of inputs describing the upper and lower bounds for the first, second, and third formant frequencies of the speech sound. Another commonly used acoustic speech feature is mel-frequency cepstral coefficients (MFCCs). A recent study [14] employed 13 MFCCs and 1 voiced/unvoiced probability along with their first and second-order derivatives as an acoustic representation of speech. These features were used as the input to the LSTM regression model which was trained to perform the acoustic-to-articulatory mapping. It has been reported that the difference between the voice of the speaker in the reference and the voice of the synthesizer drastically affected the performance of the LSTM regression model in a negative way. Partially, this is due to the MFCCs carrying extralinguistic information about the speakers’ gender, age, emotional state, etc. Thus, two contextually identical speech audio samples might have significantly different MFCCs. Even though it is advantageous in some applications, this, as shown in [14], may impose an additional challenge for finding an optimal policy or acoustic-to-articulatory mapping.

**Figure 2:**
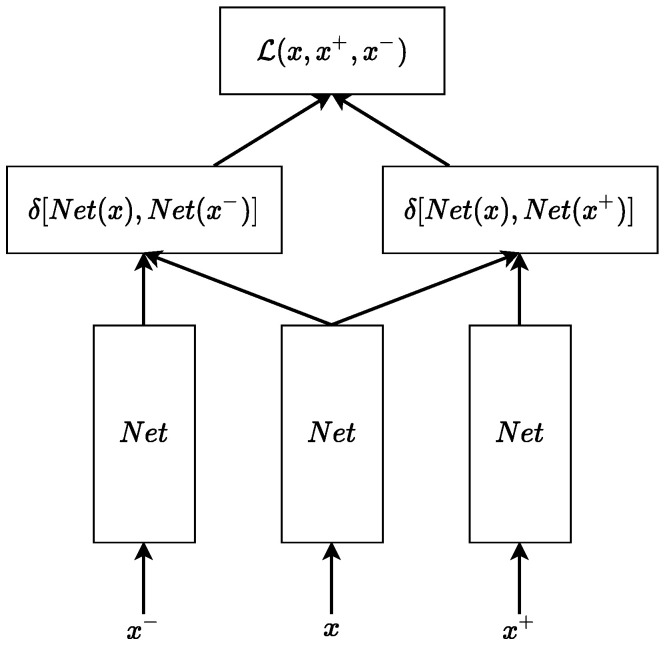
Schematic diagram of triplet loss computation in (1).

To address the problem of inter and intra-speaker variability of speech representation, the acoustic word embedding (AWE) model [22] was employed to obtain embeddings from MFCCs. These embeddings were then used as an acoustic representation of speech in the learning algorithm. The core idea of this AWE model is to train a neural network to distinguish between pairs of semantically equivalent speech audio recordings (positive pair) from pairs of speech audios corresponding to different words (negative pair). It is achieved by feeding a positive pair of MFCCs $\left(x,x^{+}\right)$ extracted frame by frame from semantically equivalent speech and a negative pair $\left(x,x^{-}\right)$ to the network. Then, the distances between outputs of the network are calculated within a positive and a negative pair and finally passed to the triplet loss function. A corresponding schematic diagram is shown in Figure 2.

The triplet loss function is defined as follows:

(1) $$\begin{array}{cc} \; & L\left(x,x^{+},x^{-}\right)=\max\left(\delta \left[Net\left(x\right),Net\left(x^{+}\right)\right]-\delta \left[Net\left(x\right),Net\left(x^{-}\right)\right]+\alpha ,0\right), \end{array}$$

where x is a matrix of MFCCs extracted from speech audio frame by frame (window size = 20 ms, stride = 10 ms), Net corresponds to the embedding neural network, δ denotes the distance function (i.e., DTW-distance utilized in the present study), and α is a margin hyper-parameter. As a result of minimizing this loss function, embeddings of similar words have a small distance and remain close to each other in the embedding space, while non-similar words occur far apart from each other and at a large distance.

Here, the AWE model was trained on the speech commands dataset in accordance with the original work [22] followed by fine-tuning on the synthetic speech dataset used as references in the vowel-to-vowel imitation task. Details of the reference dataset are presented in Section 3.3.

**Figure 3:**
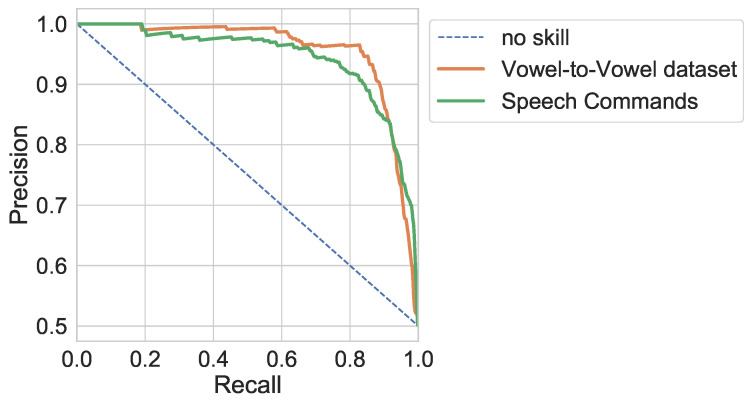
Precision vs. recall of the fine-tuned AWE model.

Precision-recall curves of the fine-tuned AWE model for word discrimination task on both speech commands and the reference vowel-to-vowel datasets are shown in Figure 3. The fine-tuned model achieved average precision (AP) of 0.943 and 0.955 for speech commands and reference vowel-to-vowel datasets accordingly.

To make the embeddings compact, the size of the final layer of the embedding neural network was set to 16 units. Aside from additional training on the reference dataset and changing the size of the output layer, there were no deviations from the training protocol described in Section 3.3. Overall, the model consisted of two stacked LSTM layers with 512 units each, followed by three consecutive fully connected layers with 256 units, 256 units, and a layer used for embedding batch-normalization with 16 units, respectively. The output of the last fully connected dropout was applied to the input of the network with a dropout rate p=0.2. An Adam optimizer [26] with a learning rate of lr=0.0005 was used during the training procedure. All models were implemented using PyTorch [27].

After training, embeddings produced using the AWE model were used as acoustic speech features, thus bringing acoustic state $S_{ac}\to R^{16}$.

### 3.2. Learning Algorithm

**Figure 4:**
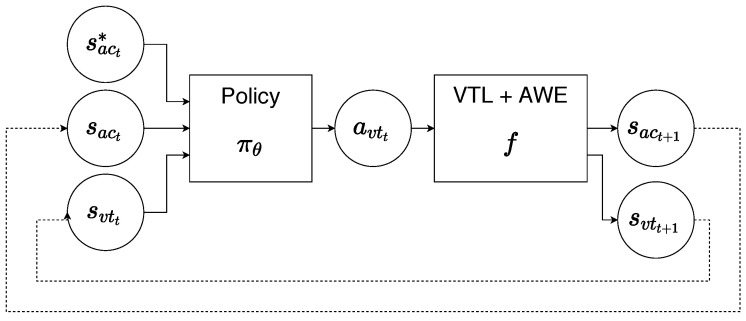
Closed-loop control of articulatory synthesizer for the imitation task.

The main objective of the proposed learning algorithm is to find the policy $\pi_{\theta }$ which controls the vocal tract of the articulatory synthesizer to imitate the reference speech. It effectively means that the resulting distance between the synthesized speech and the reference is subject to be minimized. Thus, the learning objective can be expressed as follows:

(2) $$J=\underset{\theta }{\mathrm{argmin}}\left[\delta \left(s_{ac},s_{ac}^{*}\right)\right],$$

where δ is the distance between the vector of acoustic speech features of the synthesized speech $s_{ac}$ and reference speech $s_{ac}^{*}$. A vector of acoustic speech features $s_{ac}$ is obtained using the actions produced by policy according to the scheme shown in Figure 4.

Here, at a timestep *t*, a policy $\pi_{\theta }$ receives the reference acoustic speech features $s_{ac_{t}}^{*}$ along with the current acoustic and articulatory state of the VTL model $s_{vt_{t}}$ and $s_{ac_{t}}$. Action $a_{vt_{t}}$ produced by the policy is then passed in the articulatory synthesizer VTL model which makes a transition to a new vocal tract shape denoted as $s_{vt_{t+1}}$, and synthesizes a new bit of sound corresponding to the duration of one timestep 40 ms. Acoustic embeddings $s_{ac_{t+1}}$ are then extracted from the synthesized audio using the AWE model described in Section 3.1. To improve the readability of the diagram, the VTL model and AWE model are combined into a single block. Finally, the new acoustic and articulatory states are passed into the policy forming a closed-loop control scheme.

There are numerous different approaches for optimization policy in such closed-loop scenarios. This study focuses on the particular family of methods where a model of the dynamics is learned along with the policy. One of the pioneering works in this field [28] has shown how learning a model of unknown dynamics can help to utilize a supervised learning algorithm for policy optimization. The authors employed a differentiable forward model which was learned from the mapping from actions to outcomes.The learned forward model was then used to predict the outcome of the policy’s actions and compute the corresponding error between predicted outcomes and target outcomes. Finally, the policy was optimized via a back-propagation algorithm.

The learning algorithm proposed in this study is based on the principal ideas from [28] and was adapted to the speech imitation problem. Conceptually, the proposed algorithm includes the following steps:

1. Collect a set of state-action-state transitions using the policy $\pi_{\theta }$ and synthesizer *f*.
2. Train the forward-model $\hat{f_{\psi }}\left(s,a\right)$ to minimize the prediction error on the collected data.
3. Use the learned forward model to compute the policy loss function and backpropagate the error into the policy through the forward model.
4. Update policy parameters.
5. Go to 1.

The learning algorithm depicted in Algorithm 1 is an iterative process where each step conceptually consists of data collection, updating the forward model, and updating the policy.

Data collection was accomplished by executing the closed-loop shown in Figure 4. Each state–action–state transition then was stored in the experience replay buffer $D=\{\left(s_{t}^{*},s_{t},a_{t},s_{t+1}\right)\}$. Note that transitions from previous steps were kept in the replay buffer, thus increasing the buffer’s size and enabling the more generalized training of the forward-model.

One important addition to the data collection was the introduction of an early stopping condition for the control loop. Normally, the execution would stop when the duration of the synthesized speech reached the duration of the reference, indicating the end of the episode. In this study, an additional stop condition was included which terminated the episode when the DTW distance between the synthesized speech and the reference exceeded a certain threshold defined by the hyper-parameter ϵ. Effectively, this stop condition constrains the collected acoustic data by keeping it within a certain distance limit from the reference acoustics and increases its utility for training the forward model which leads to a higher sample efficiency of the learning algorithm.

The forward-model $\hat{f_{\psi }}$ is a neural network that learns a prediction $\hat{s_{t+1}}$ of the next state $s_{t+1}$ given the current state $s_{t}=\left[s_{vt_{t}},s_{ac_{t}}\right]$ and action $a_{t}$:

(3) $$\hat{s_{t+1}}=\hat{f_{\psi }}\left(s_{t},a_{t}\right)$$

**Figure 5:**
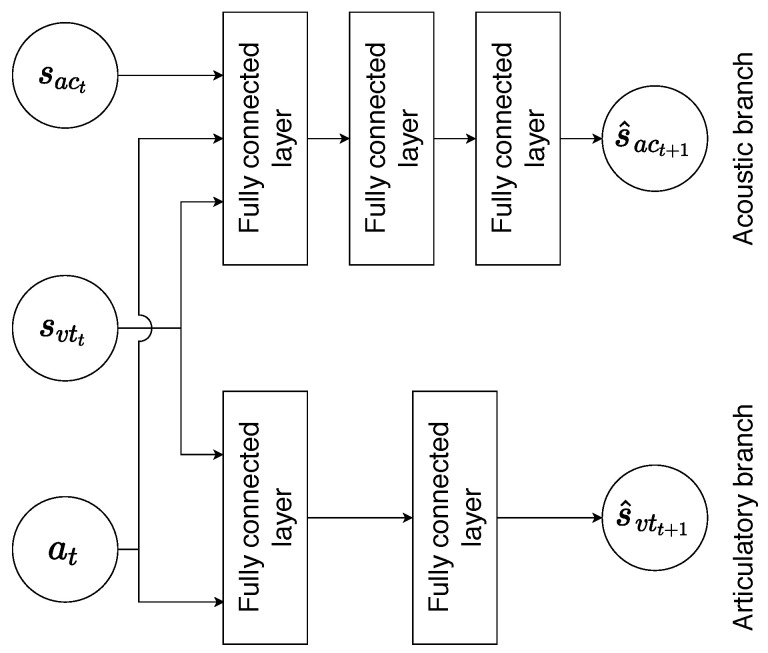
Structure of the forward-model neural network with two separate branches.

The structure of the neural network used for the forward model is shown in Figure 5. It has two separate branches for predicting articulatory and acoustic states, respectively. The articulatory branch takes the current articulatory state $s_{vt_{t}}$ and action $a_{t}$ as an input and predicts the next state ${\hat{s}}_{vt_{t+1}}$. Due to the relatively simple dynamics of the articulatory movements, this branch consists of just two stacked fully connected layers with 256 and 23 units each.
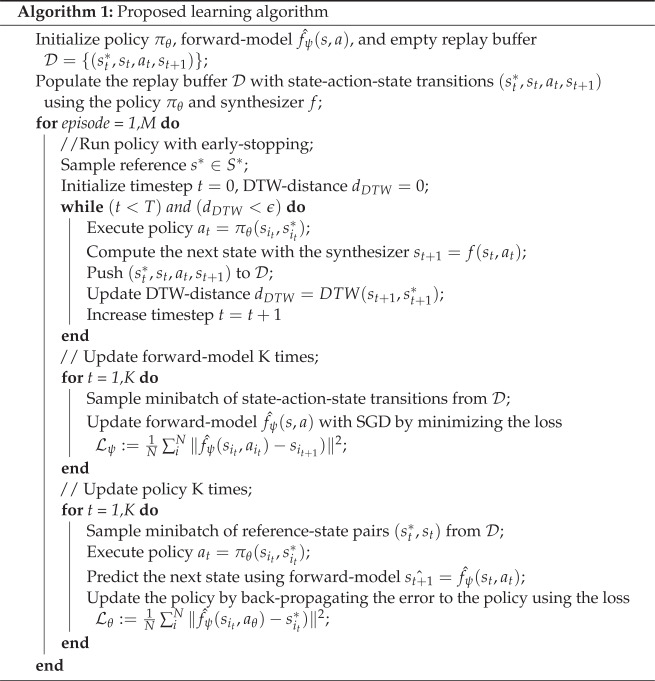


The acoustic branch has three stacked fully connected layers with 512, 256, and 16 units respectively, where the output of the last layer is a 16-dimensional vector that matches the dimension of the acoustic state. The acoustic branch receives both articulatory $s_{vt_{t}}$ and acoustic $s_{ac_{t}}$ states as inputs along with the action $a_{t}$, and predicts the next acoustic state ${\hat{s}}_{ac_{t+1}}$. The RELU activation function was applied to the outputs of all hidden layers.

This network was trained via the stochastic gradient descent (SGD) algorithm with a learning rate lr=0.0005 and using the following prediction loss function:

(4) $$L_{\psi }:=\frac{1}{N}\sum_{i}^{N}∥\hat{f_{\psi }}\left(s_{i_{t}},a_{i_{t}}\right)-s_{i_{t+1}}{)∥}^{2}$$

where the state–action–state transitions $\left(s_{i_{t}},a_{i_{t}},s_{i_{t+1}}\right)$ are drawn from the experience replay buffer D, N is the number of samples in the mini-batch, and ψ are the parameters of the neural network. Minimizing the loss function $L_{\psi }$ ensures that the forward model learns to predict the next state of the system and, thus, serves as an approximation of the actual VTL model. It allows us to use the forward model for updating the policy.

The policy was represented by a neural network that was trained to predict the best action (i.e., how to move articulators of the vocal tract) for imitating the reference. Thus, as shown in Figure 4, the policy takes acoustic reference $s_{ac_{t}}^{*}$ and both current articulatory $s_{vt_{t}}$ and acoustic states $s_{ac_{t}}$ as the inputs. Unlike the branching structure of the forward model, the policy network was implemented as a plain stack of three fully connected layers with 256, 128, 23 units, respectively. The RELU activation function was applied to the outputs of all layers excluding the final layer. The final layer of the network outputs the action *a*, a 23-dimensional vector that directs the movement of vocal tract articulators.

Similar to learning the forward model, the policy network was trained via the SGD algorithm with a learning rate lr=0.0005. The loss function used for the optimization was given as:

(5) $$L_{\theta }:=\frac{1}{N}\sum_{i}^{N}∥\hat{f_{\psi }}\left(s_{i_{t}},a_{\theta }\right)-s_{i_{t}}^{*}{)∥}^{2}$$

where the current state $s_{i_{t}}$ and corresponding acoustic reference $s_{i_{t}}^{*}$ are drawn from the replay buffer D and action $a_{\theta }$ is obtained using the current policy $a_{\theta }=\pi_{\theta }\left(s_{i_{t}},s_{i_{t}}^{*}\right)$.

**Figure 6:**
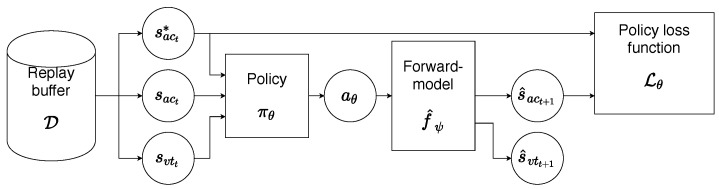
Computing the policy loss function using the forward-model.

The computation of the policy loss function $L_{\theta }$ is shown in Figure 6. First, the pair of state and reference were sampled from the replay buffer D. It is important to note how the action of the corresponding experience is ignored. Instead, a new action $a_{\theta }$ is obtained using the current policy $\pi_{\theta }$. Then, the forward-model predicts the acoustic outcome $\hat{f_{\psi }}\left(s_{i_{t}},a_{\theta }\right)$ of applying this action which is finally passed into the loss function. Substituting the actual vocal tract model with the learned forward model allows us to backpropagate the computed error back to the policy and update its parameters θ using the gradient descent algorithm.

Updating the policy finalizes the learning iteration step. Upon this, the algorithm starts a new iteration by collecting new data using the updated policy. One specific feature of this online learning scenario is that the iterative training of both the forward model and the policy allows to effectively bootstrap each other over the learning course. The data collected using the policy is used to update the forward model, thus, leading to a better approximation of the true speech production dynamics. The improved forward model allows for more accurate computing of the policy loss function and updating the policy parameters, hence improving its performance.

### 3.3. Reference Dataset

For the purpose of this study, a set of reference speech samples was synthesized using the same VTL articulatory model with predefined vocal tract shapes for five English cardinal vowels: [a], [e], [i], [o], [u]. Each sample in the dataset is a smooth transition between a pair of these vowels. In total, the reference set contained 25 different vowel-to-vowel syllables. The reason for employing this set of sounds is that the production of cardinal vowels requires extreme positioning of the tongue, implying that the learning procedure will involve extensive exploration of the articulatory space.

To imitate natural speech diversity, a small amount of Gaussian noise was injected into the articulatory movements. Speaking tempo and timings of transition between individual phones were also varied across references so that the transition from one vowel to another lasted from 0.1 to 0.6 s. All these modifications were implemented to reflect the fact that speech naturally varies in terms of articulation and timing. After synthesis, the quality and intelligibility of all audio samples were manually assessed and samples with unintelligible speech were excluded from the dataset.

Each vowel-to-vowel transition was synthesized 50 times, making the total number of reference samples in the dataset 1000. A few examples of the reference samples can be found in the video materials included in this study which can be accessed via Supplementary Materials Video.

### 3.4. Evaluation Metrics

Since the goal of the policy is to produce a sound similar to the reference sound, an evaluation of the learned policy was performed in terms of acoustic similarity. One important aspect of methods evaluating acoustic similarity algorithms is the representation of the speech sound. Commonly, MFCCs are used for this purpose. In addition to MFCCs, AWEs were used as speech representations in this study.

**Figure 7:**
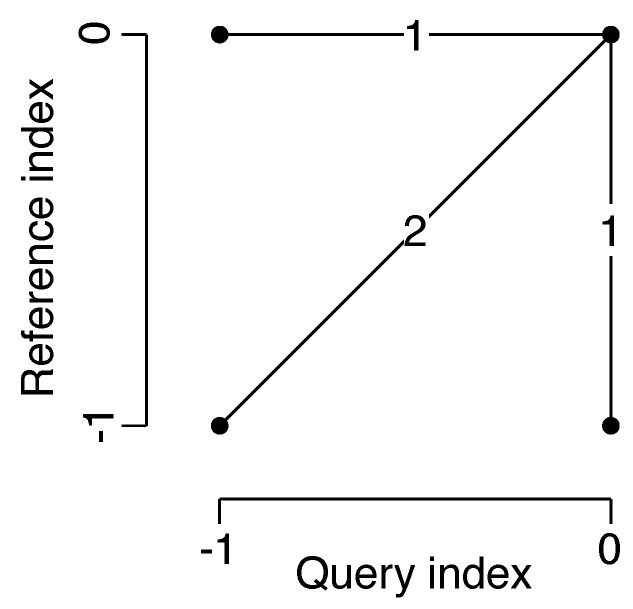
*Symmetric2* step-pattern used in DTW algorithm.

Once the representations of two speech samples are obtained, they need to be matched. The most common algorithm is dynamic time warping (DTW) which uses dynamic programming to find the optimal alignment between two temporal sequences. In this study, the DTW-distance was used to measure the similarity between two speech samples with a lower distance corresponding to more similar samples. A DTW algorithm was implemented with commonly used Euclidean distance as a local cost function and a *symmetric2* step-pattern, shown in Figure 7. The other algorithm used for evaluation in this study is cross-correlation, which aligns two time series of variable lags. Taking the maximum value of the alignment gives a similarity value for the two time series, with higher values corresponding to higher similarity. Overall, four methods **$DTW_{mfcc}$**, **$DTW_{awe}$**, **$CC_{mfcc}$**, **$CC_{awe}$** for evaluating the policy were applied in this study by combining speech representations (MFCCs, AWEs) and different metrics (DTW, cross-correlation).

In order to analyze the performance of the learned policy in terms of the proposed metrics, the average maximum of cross-correlation and DTW-distance was computed between pairs of samples of matching class and non-matching classes (samples from different classes) in the reference dataset. Table 2 demonstrates the mean and standard deviation of each metric for both pair types. It can be seen that for both metrics **$DTW_{mfcc}$** and **$DTW_{awe}$**, the DTW-distance is significantly bigger for pairs with non-matching classes than for pairs with matching classes which indicates that DTW-based metrics successfully capture the similarity between samples in the reference dataset. Cross-correlation metrics **$CC_{mfcc}$** and **$CC_{awe}$** were also able to differentiate between the pairs with matching and non-matching classes with higher values corresponding to higher similarity. Overall, the values presented in Table 2 were used in the evaluation of the learned policy in Section 5 as an indication of successful learning. Further detailed analysis of the proposed metrics is included in Appendix A.

## 4. Experiments

To evaluate the performance of the proposed algorithm, the policy was trained to imitate vowel-to-vowel references drawn from the reference dataset presented in Section 3.3. The algorithm was implemented in python using Pytorch [29]. The number of learning iterations was fixed to 15,000. All training runs have been executed on a machine with a single NVIDIA Quadro P6000 and took approximately 5 h per run.

According to Section 3.4, four metrics were used to evaluate the performance of the policy by comparing the reference speech sample and synthesized speech. Thus, for DTW-based metrics **$DTW_{mfcc}$** and **$DTW_{awe}$**, lower values correspond to a better-performing policy. Accordingly, for cross-correlation metrics **$CC_{mfcc}$** and **$CC_{awe}$**, higher values indicate better performance of the policy. Optimal values for all four metrics were obtained by analyzing the pairs with matching classes in the reference dataset and are shown in the column *Matching classes* in Table 2.

As mentioned in Section 3.3, reference sound samples were synthesized using the VTL model by manually adjusting the vocal tract shape to produce the desired vowel-to-vowel speech sound. This approach allows us to access the actual vocal shape corresponding to the reference speech sound and extend the reference representation while learning the policy. Thus, alongside the original scenario, three additional experiments were performed using different reference representations:

- **Acoustic reference (ac)** (original scenario). In this case, the policy had access only to the acoustic features of the reference samples $s_{ac}^{*}$
- **Acoustic + articulatory reference (ac + vt)**. The policy had access to the full reference state meaning both acoustic and articulatory states $s^{*}=\left[s_{ac}^{*},s_{vt}^{*}\right]$
- **Acoustic + partially observable articulatory reference (ac + partial vt)**. The policy had access to the acoustic features of the reference as well as six articulatory parameters defining the positioning of lips, jaw, and hyoid bone. Namely, the parameters were: HX, HY, JX, JA, LP, LP. The idea behind selecting these parameters is that they are visually accessible and can be derived by analysis of the facial movements of the speaker uttering the reference sound.
- **Articulatory reference (vt)**. The policy observed only the articulatory state of the reference $s_{vt}^{*}$

By comparing the results of these four experiments it was possible to determine if the learning can benefit from additional information about the vocal tract shape. To some extent, the **acoustic + partially observable articulatory reference** scenario relates to how children develop speaking skills through imitation. While listening to someone speaking, a child usually gets some visual clues about the speaker’s vocal tract shape by focusing their visual attention on the lips movement and jaw articulation, and elevation of the hyoid bone. Whereas, the **articulatory reference** scenario corresponds to the primary goal of the study which is the policy learning based exclusively on the acoustic reference without relying on any visual clues of the articulatory movements.

## 5. Results and Discussion

The results of the experiments described in Section 4 are shown in Table 3. The performance of the learned policies trained in four different training scenarios **vt**, **vt + ac**, **ac + partial vt**, **vt** was evaluated using the following metrics: $DTW_{mfcc}$, $DTW_{awe}$, $CC_{mfcc}$, $CC_{awe}$. The last row in Table 3 depicts the acoustic similarity between speech samples of the same class from the reference dataset and indicates the values at each vowel-to-vowel imitation task that can be considered solved. For DTW-based metrics $DTW_{mfcc}$ and $DTW_{awe}$, the task is solved when the model achieves DTW-distances equal or below 0.22 and 0.14 respectively. For $CC_{mfcc}$ and $CC_{awe}$, the model needs to achieve maximum cross-correlation 0.89 and 0.88 to solve the task.

The training scenario **vt** included only articulatory features as the reference representation. As can be seen from Table 3, the learned policy successfully solved the task according to all four metrics used for evaluation. We also observed that training in this scenario had the fastest convergence and policy was able to imitate reference sounds after a few hundred training iterations. The high performance and fast convergence can be explained by the fact that this scenario imposes a fairly simple problem to solve where the policy needs to learn how to control a vocal tract while being able to observe the reference vocal tract shape.

In scenario **vt + ac**, policy had full access to the reference including both the articulatory **vt** and acoustic **ac** states. As a result, the learned policy was able to not only solve the task but also demonstrated the best performance according to $DTW_{awe}$ and $CC_{awe}$ metrics. The policy has achieved $DTW_{awe}=0.10$ which is substantially smaller than the distance measured between similar samples from the reference dataset. It indicates that the policy managed to reproduce the reference sounds acoustically more similar than other samples from the dataset of the same class.

Despite the remarkably high performance, these two training scenarios should be taken with a grain of salt since they effectively transformed the original unsupervised imitation task into the supervised domain. The practical outcome of these two experiments was that they confirmed the hypothesis that by letting the policy observe the vocal tract shape of the reference sound, a significant increase of the learning convergence rate and an improvement of the final performance of the learned policy can be achieved.

The third training scenario **ac + partial vt** corresponded to the case when, along with the acoustic features of the reference, the policy had access to the visually accessible articulators. As discussed in Section 4, this scenario showcases the learner attempting to imitate the external speaker while looking at the speaker’s face and observing the movements of lips, jaw, and hyoid bone. In this scenario, the policy had access only to six articulatory parameters of the reference, which accounts for a fairly small fraction of the total number of vocal tract parameters equal to 23. As shown in Table 3, the policy performed worse than in the first two training scenarios **ac + partial vt** and **vt**. It solved the task only according to the $CC_{awe}$ achieving the value of 0.88. However, according to the remaining metrics, the policy’s performance was close to solving the task. For example, in the case of the $DTW_{awe}$, the policy achieved a distance score of 0.15 which is just 0.01 bigger than the average distance between similar sounds from the reference dataset.

The final training scenario **ac** corresponds to the primary imitation task when the policy needed to learn how to control a vocal tract while observing only the acoustic features of the reference speech sound. We observed that training in this scenario was the least stable and the policy’s error fluctuated throughout the training significantly. This observation is in accordance with the general observation of the model-based approaches proven to be highly unstable and sensitive to hyper-parameters. Moreover, our preliminary experiments have shown that training the policy in this scenario without early stopping never converged to even a sub-optimal solution. In addition, we failed to successfully train the policy in the case when MFCCs were used as acoustic representations instead of the proposed AWE embeddings. When early-stopping and AWE embeddings were employed, the final $DTW_{awe}$-distance of the learned policy achieved a value of 0.18. Interestingly, in case of MFCC-based metrics $DTW_{mfcc}$ and $CC_{mfcc}$, the performance of the policy was comparable to the others and even achieved $DTW_{mfcc}=0.23$ which is lower than the policy trained in **ac + partial vt** scenario. However, AWE-based metrics showed that the **ac** policy performed significantly worse compared to others including **ac + partial vt**. This discrepancy between MFCC and AWE-based results may indicate that AWE-based metrics are more sensitive than their MFCC-based analogues which can be advantageous in some applications. Overall this observation leads to an interesting direction for future research aiming to analyze the difference between these metrics and their suitability as an acoustic similarity measure.

**Figure 8:**
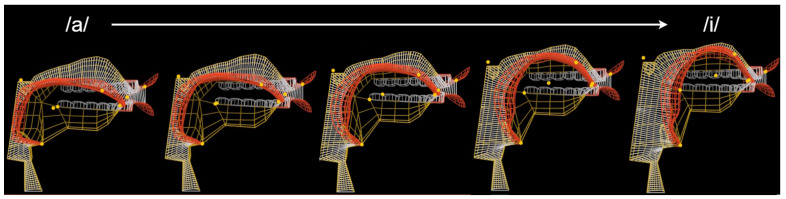
Manually controlled vocal tract while synthesizing/ai/sound.

**Figure 9:**
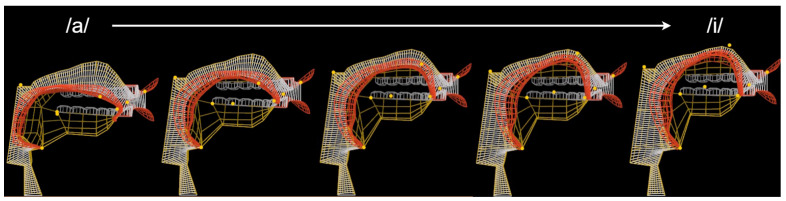
Vocal tract controlled by the learned policy while synthesizing/ai/sound.

A video showing the progress of the learning in the **ac** training scenario can be accessed via Supplementary Materials Video. In addition, snapshots of the vocal tract controlled by the learned policy while attempting to imitate the /ai/ sound are shown in Figure 8 and Figure 9.

Visual analysis of the vocal tract shapes, presented in Figure 8 and Figure 9, demonstrates that the policy successfully learned that /ai/ sound is produced by raising the tongue and pushing it to the front while the tip of the tongue can be lowered just behind the bottom front teeth. A Supplementary Materials Video allows comparing the reference audios with the synthesized speech. By analyzing these results, it can be concluded that even though, the policy did not learn how to perfectly imitate the reference, it achieved a fairly close match both visually and acoustically.

## 6. Conclusions

This study investigated the applicability of a model-based reinforcement learning method in the domain of articulatory synthesis for a vowel-to-vowel imitation task. The proposed algorithm was based on iterative learning of the forward model, followed by updating the policy. As a result, the policy was trained to imitate the reference sound closely. Early stopping was employed during the data collection stage to stabilize the training. Along with using acoustic word embeddings as acoustic features, these additions to the proposed algorithm have shown to be crucial for successful training.

There are several directions for future research. The suggestion is to move towards general articulatory speech synthesis and learn how to imitate full words. Another important aspect not covered in this study is to investigate how robust the proposed algorithm is for different voices in the reference samples. The algorithm has no explicit constraint for the synthetic voice to match the reference’s voice. Further, the evaluation metric and acoustic features were designed to allow this mismatch. This assumption, however, needs to be verified through additional experiments. Finally, there is clearly room for improvement regarding the stability of convergence. Thus, one of the potential directions for future research is to further investigate the possibility of improving this aspect of the algorithm.

## Figures and Tables

**Table 1:** Control parameters of the vocal tract model (adapted from [7]). For each parameter, the value range and the unit is given. Parameters without a unit specify relative values.

Index	Name	Description	Min	Max	Unit
0	HX	Horiz. hyoid position	0.0	1.0	
1	HY	Vert. hyoid position	−6.0	−3.5	cm
2	JX	Horizontal jaw displacement	−0.5	0.0	cm
3	JA	Jaw angle	−7.0	0.0	deg
4	LP	Lip protrusion	−1.0	1.0	
5	LD	Vert. lip distance	−2.0	4.0	cm
6	VS	Velum shape	0.0	1.0	
7	VO	Velic opening	−0.1	1.0	
8	TCX	Tongue body center X	−3.0	4.0	cm
9	TCY	Tongue body center Y	−3.0	1.0	cm
10	TTX	Tongue tip X	1.5	5.5	cm
11	TTY	Tongue tip Y	−3.0	2.5	cm
12	TBX	Tongue blade X	−3.0	4.0	cm
13	TBY	Tongue blade Y	−3.0	5.0	cm
14	TRX	Tongue root X	−4.0	2.0	cm
15	TRY	Tongue root Y	−6.0	0.0	cm
16	TS1	Tongue side elevation 1	−1.4	1.4	cm
17	TS2	Tongue side elevation 2	−1.4	1.4	cm
18	TS3	Tongue side elevation 3	−1.4	1.4	cm
19	TS4	Tongue side elevation 4	−1.4	1.4	cm
20	MA1	Min. area tongue back region	0.0	0.3	cm${}^{2}$
21	MA2	Min. area tongue tip region	0.0	0.3	cm${}^{2}$
22	MA3	Min. area lip region	0.0	0.3	cm${}^{2}$

**Table 2:** Average DTW-distance and maximum of cross-correlation between samples in the reference dataset.

Metric	Matching Classes		Non-Matching Classes	
	Mean	Std	Mean	Std
** $DTW_{mfcc}$ **	0.22	0.02	0.30	0.02
** $DTW_{awe}$ **	0.14	0.02	0.22	0.03
** $CC_{mfcc}$ **	0.89	0.01	0.85	0.02
** $CC_{awe}$ **	0.88	0.02	0.78	0.05

**Table 3:** Evaluation results for each training scenario.

Training Scenario	Metrics			
	${\mathrm{DTW}}_{\mathrm{mfcc}}$	${\mathrm{DTW}}_{\mathrm{awe}}$	${\mathrm{CC}}_{\mathrm{mfcc}}$	${\mathrm{CC}}_{\mathrm{awe}}$
vt	0.21	0.14	0.89	0.88
ac + vt	0.22	0.10	0.89	0.91
ac + partial vt	0.24	0.15	0.88	0.88
ac	0.23	0.18	0.88	0.84
optimal	0.22±0.02	0.14±0.02	0.89±0.01	0.88±0.02

**Figure A1:**
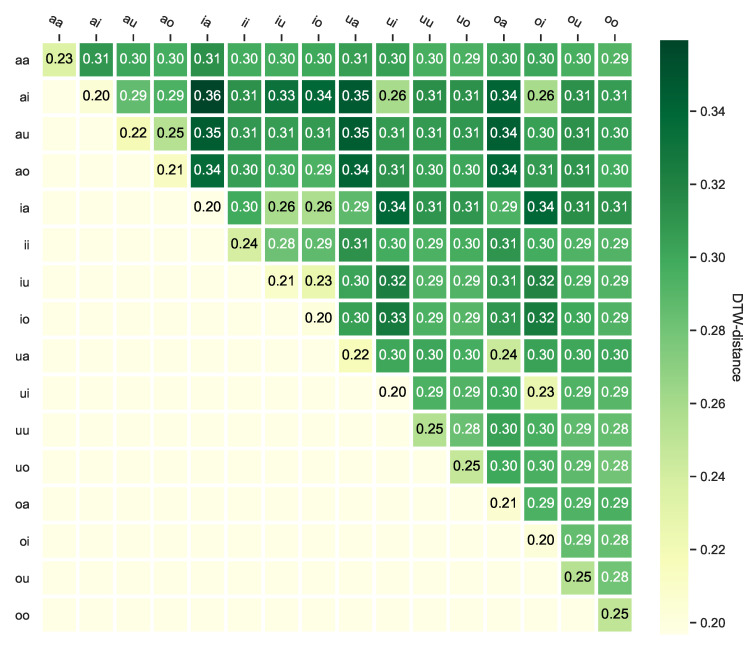
$DTW_{mfcc}$ calculated between samples from reference dataset. Lower values correspond to acoustically more similar samples.

**Figure A2:**
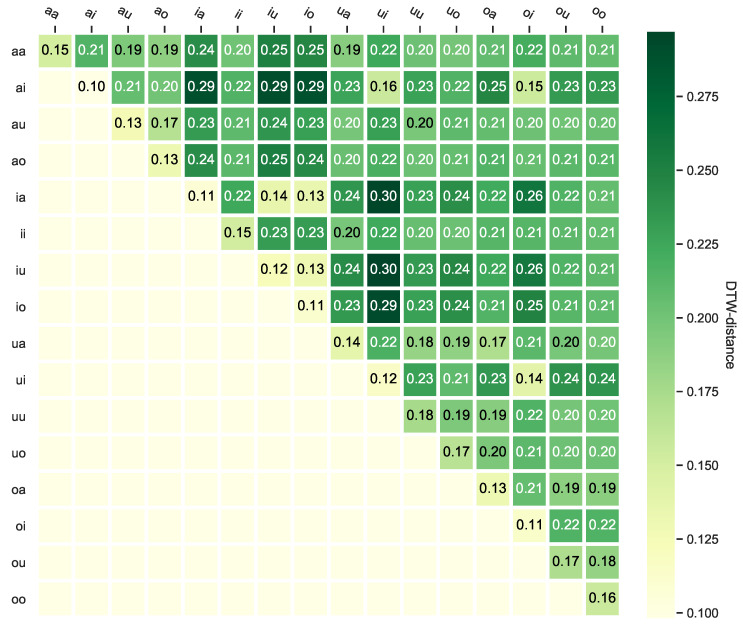
$DTW_{awe}$ calculated between samples from reference dataset. Lower values correspond to acoustically more similar samples.

**Figure A3:**
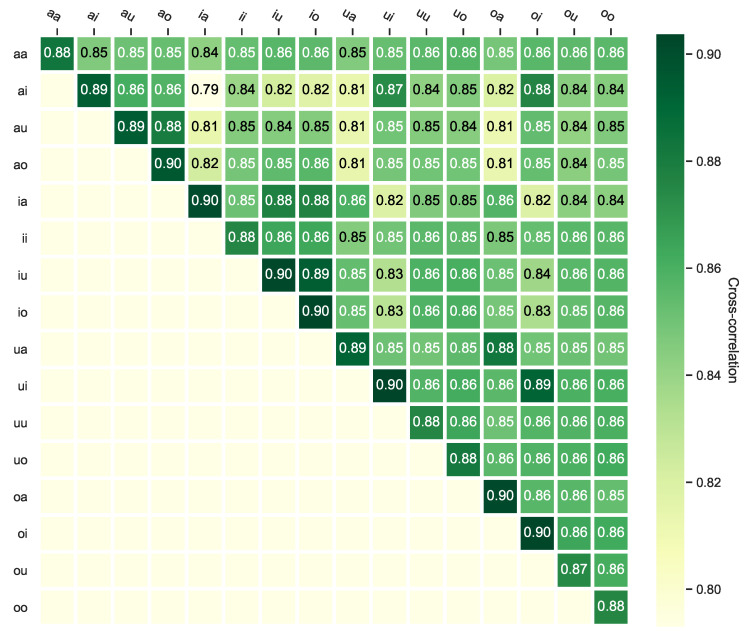
$CC_{mfcc}$ calculated between samples from reference dataset. Higher values correspond to acoustically more similar samples.

**Figure A4:**
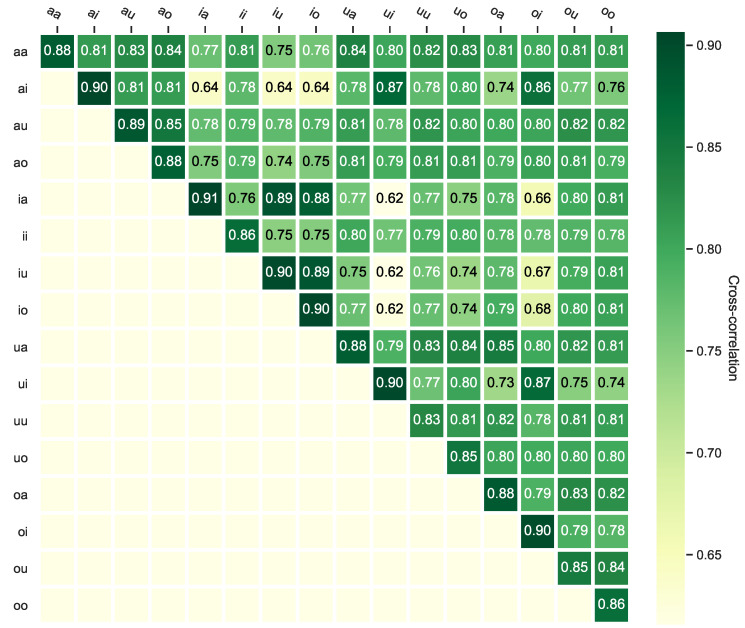
$CC_{awe}$ calculated between samples from reference dataset. Higher values correspond to acoustically more similar samples.

## Data Availability

Not applicable.

## Supplementary Materials

The following supporting information can be downloaded at: https://youtu.be/elhHpZgIyIU, Self-learning algorithm for speech production by a simulated vocal tract. (accessed on 1 January 2023).

## Appendix A

This appendix contains Supplementary Material for Section 3.4 and expands Table 2. Column *Matching classes* in Table 2 shows the average metric value calculated between pairs of samples from the same class (e.g., /ai/ and /ai/). Column *Non-matching classes* in Table 2 shows the average metric value calculated between all other pairs of samples. Figure A1–Figure A4 provide a detailed overview of the metrics values calculated between each possible pair of samples from the reference dataset. Values on the main diagonal correspond to the sample pairs of the class (*Matching classes*). Values in the upper triangle except for the ones on the main diagonal correspond to the *Non-matching classes*.
